# An Analysis of Biopsies for Suspected Skin Cancer at a Tertiary Care Dermatology Clinic in the Western Cape Province of South Africa

**DOI:** 10.1155/2020/9061532

**Published:** 2020-01-27

**Authors:** Johann de Wet, Minette Steyn, Henry F. Jordaan, Rhodine Smith, Saskya Claasens, Willem I. Visser

**Affiliations:** ^1^Division of Dermatology, Department of Medicine, Tygerberg Academic Hospital, Stellenbosch University, Cape Town, South Africa; ^2^Faculty of Medicine and Health Sciences, Stellenbosch University, Cape Town, South Africa; ^3^Division of Epidemiology and Biostatistics, Department of Global Health, Stellenbosch University, Cape Town, South Africa

## Abstract

**Background:**

Skin cancer is a growing health concern worldwide. It is the most common malignancy in South Africa and places a large burden on the public healthcare sector. There is a paucity of published scientific data on skin cancer in South Africa.

**Objectives:**

To report the findings of biopsies performed in patients with suspected skin cancer attending the Tygerberg Academic Hospital (TAH) Dermatology outpatient department (OPD) in the Western Cape Province of South Africa. *Methodology*: A retrospective chart review identified all patients who underwent a biopsy for a suspected skin cancer diagnosis between September 2015 and August 2016 at the TAH dermatology OPD.

**Results:**

A total number of 696 biopsies from 390 participants were identified, of which 460 were histologically confirmed as malignant lesions. The proportion of clinically suspected skin cancers that were histologically confirmed as cancer was 68%. The most commonly occurring malignancies were basal cell carcinoma (BCC) (54.8%), squamous cell carcinoma (SCC) (18.9%), squamous cell carcinoma in-situ (SCCI) (8.0%), Kaposi's sarcoma (KS) (6.7%), malignant melanoma (MM) (6.1%), and keratoacanthoma (KA) (4.6%). The number needed to treat (NTT) for all cancers diagnosed and for MM was 1.5 and 4 respectively. BCC (89.3%) and KS (67.7%) was the most common skin cancer in the white and black population respectively. The ratio of BCC to SCC was 2.03.

**Conclusion:**

This study provides valuable scientific data on the accuracy of skin cancer diagnosis, distribution and patient demographics in the Western Cape Province of South Africa, on which further research can be based. The study highlights the burden of skin cancer on this specific population group and calls for standardised reporting methods and increased surveillance of skin cancers.

## 1. Introduction

Skin cancer is the most common malignancy worldwide and is typically divided into MM and non-melanoma skin cancer (NMSC) [1]. The term NMSC encompasses BCC and SCC but also includes rarer types of skin cancer such as Merkel cell carcinoma and KS [2]. The worldwide incidence of NMSC and MM has been steadily increasing [3–7].

Most of the studies on NMSC have focussed on white populations in Europe, the United States (US), and Australia with limited data for other skin types in regions such as Africa [1].

The South African population is at a particularly high risk of skin cancer due to the country's geographical position and level of ultraviolet radiation (UVR) as well as the increased sun exposure due to occupational and recreational activities [8, 9]. While incidence rates for NMSC in South Africa remain high, rates are often grossly underreported due to incomplete case reporting to the National Cancer Registry (NCR). It is estimated that 25.4% of all cancers diagnosed in men in South Africa in 2014 were BCC while 10.9% were SCC [10]. In females, the numbers were 18.6% and 7.1% for BCCs and SCCs respectively [10]. This shows an increase when compared to numbers from 2009 [11]. Although data on MM incidence on the African continent remain scarce, a recent study showed the overall incidence of MM for South Africa to be 2.7 per 100 000, with the incidence in the white population being significantly higher at 23.2 per 100 000 [12]. MM in darker skin types often presents as acral melanoma (AM), a rare distinct variant of MM that arises from the palms, soles, and nail apparatus and is associated with a poorer prognosis [13–15].

While skin type and UVR play a large role in determining the risk of developing skin cancer, there are also many other factors at play, including immunosuppression. The prevalence of Human Immunodeficiency Virus (HIV) in South Africa was estimated at 12.7% in 2016 making the total number of people living with HIV (PLHIV) approximately 7.03 million [16, 17]. Despite the rollout of the antiretroviral therapy (ART) programme in South Africa in 2004 the risk of KS amongst PLHIV remains elevated even in the ART era [18]. Omland et al. also observed a 2-fold increased risk of BCC and a 5-fold increased risk of SCC in PLHIV compared with the background population [19].

The increasing incidence of skin cancers places major financial strain on South Africa's already overburdened public healthcare system. A recent study estimated the annual cost of skin cancer treatment in the country to be ZAR 92.4 million ($15.7 million) with a further ZAR 45.1 million ($7.7 million) spent on the workup of suspicious lesions that were ultimately diagnosed as benign [20].

The above emphasises the growing need for accurate data capturing of skin cancer in South Africa to promote and assist research, as well as to increase awareness regarding prevention. This study aimed to address the gap in the availability of data and can pave the way for other research to be done on skin cancers at other similar clinics in South Africa.

## 2. Objectives

The primary objective was to assess the number of biopsies performed and determine the frequency and spectrum of histologically confirmed skin cancer. The secondary objectives were to (i) describe the demographics of patients diagnosed with skin cancer, (ii) duration and location of skin lesions biopsied and (iii) determine the accuracy of skin cancer diagnosis by calculating the number needed to treat (NNT).

## 3. Materials and Methods

A retrospective study of a descriptive nature was conducted. It consisted of patients who received a biopsy specifically to diagnose skin cancer (MM and NMSC) at the dermatology OPD at TAH between 1st September 2015 and 31st August 2016. The exclusion criteria entailed the following: (i) patients under 18 years of age (ii), biopsies done for primary cutaneous T-cell lymphoma. Patients were identified using the biopsy registry of the dermatology OPD, TAH. These biopsies were performed by registrars and consultants in the field of dermatology. Demographic, clinical, and histological data were collected from pathology reports from the National Health Laboratory Service at TAH. A hot-deck imputation method was used for subjects where ethnicity was not indicated. Subjects were assigned to a certain ethnic group by comparing their surnames with a reference database of approximately 1.4 million surnames of known ethnicity. This method was constructed for the NCR as part of a Statistical Analysis Software program by the Data Management and Statistical Analysis Unit of the University of the Witwatersrand [21]. The NNT for all cancers refers to the number of biopsies performed to make the diagnoses of one skin cancer (all biopsies performed for skin cancer divided by the number of skin cancers histologically confirmed). We calculated the NNT for MM, as the total number of pigmented lesions biopsied (where the clinician indicated melanoma as a provisional diagnosis) divided by the number of histologically confirmed MM. Stata version 14 was used for data analysis. The analysis was of a descriptive nature. A biostatistician was consulted to assist with data analysis.

The study was performed in accordance with ethical principles in the Declaration of Helsinki and Good Clinical Practice. It was approved by the Health Research Ethics Committee of the Faculty of Medicine and Health Sciences, University of Stellenbosch (HREC/REF: U16/10/028). All data collected was held under the provisions of the 2013 Protection of Personal Information Act (SA) and stored in secure manual and electronic files.

## 4. Results

A total number of 1 444 biopsies were performed over a one-year period (September 2015–August 2016) at the dermatology OPD at TAH, of which 696 biopsies, from 390 unique participants, met the inclusion criteria.

Of the 696 biopsies performed on clinically suspected skin cancers, 460 (66.1%) were histologically confirmed skin cancers, 216 (31%) were reported as benign lesions and in 20 cases (2.9%) a histological diagnosis could not be made (Table 1). The percentage of lesions tested for skin cancer and confirmed histologically was 68%, yielding an NNT of 1.5 for all skin cancers. The NNT for melanoma was 4. The baseline demographics of participants where skin cancer was confirmed histologically is summarised in Table 2.

In 86.3% of the histologically confirmed skin cancers the exact histological diagnosis was included in the provisional differential diagnosis by the clinician.

The commonest method of tumor sampling was punch biopsy (50%) followed by curette (10.1%), excision biopsy (9.1%), and then shave biopsy (1%) while the method used was unspecified in 29.9% of biopsies done.

The most frequently biopsied body site was the face followed by the extremities and trunk (Table 3).

The most commonly occurring malignancies were BCC (54.8%), SCC (18.9%), SCCI (8%), KS (6.7%), MM (6.1%), KA (4.6%), and other malignant (0.9%). The ratio of BCC to SCC was 2.03.

Most lesions confirmed as malignancies were present between 1 and 6 months at the time of biopsy as reported by the patient.

The most frequent skin cancer per ethnic group is summarised in Table 4 and the age and gender distribution per skin cancer in Table 5.

## 5. Discussion

During the study period 48.2% of all biopsies performed at the dermatology OPD at TAH were aimed at confirming or excluding a diagnosis of skin cancer. This finding highlights the burden that skin cancer places on a tertiary dermatology clinic in the public health care system in the Western Cape Province of South Africa. The number may even be an underestimation of the prevalence of skin cancer in the study population as NMSC are occasionally diagnosed clinically and treated without a biopsy [1].

Of all skin lesions biopsied 45.5% of lesions occurred on the face reflecting the propensity for skin cancer to affect sun-exposed sites. A recent British Association of Dermatologists National Audit on NMSC Excision also reported excisions from the head and neck to account for the majority (56.7%) of cases [22].

With regards to the demographics of subjects with a confirmed diagnosis of skin cancer it is not surprising that the majority of patients were identified as white patients, as this group will be most at risk of developing skin cancer [2, 12] (Table 2). The mean age for patients with confirmed skin cancer was 65.68 and in keeping with previous reports on age for MM and NMSC that showed people over the age of 60 to have a higher incidence of skin cancer [2, 12].

In our study cohort the most common malignancies in descending order were BCC, SCC, SCCI, MM, KS, and KA. This is in keeping with international data on skin cancer, which reports BCC as the most common cutaneous malignancy [1, 2, 23]. However, studies conducted in sub-Saharan Africa concluded that SCC was the most common malignancy followed by KS. The largest review reported SCC and KS to constitute 44% and 25% respectively of the cutaneous malignancies and BCC's only 7% [24, 25]. Interestingly a recent study done in the Northern Cape Province of South Africa also found that 45.4% of skin cancers diagnosed were SCC while only 27.8% were BCC [26]. These differences in skin cancer frequency can most likely be attributed to the difference in population distribution in the Western Cape when compared to that of other sub-Saharan African countries and other parts of South Africa. The largest population group residing in the Western Cape identifies as mixed ancestry with the population distribution being as follows: mixed ancestry (47.5%), black (35.7%), white (16.0%), and Indian/Asian (0.8%) [27]. In contrast the largest population group in other parts of South Africa and sub-Saharan Africa will identify as black. Skin cancer prevalence differs in populations of different skin types. BCC is the most common skin cancer in white, Hispanic, and Asian (Japanese and Chinese) populations, as is reflected in this current study, while SCC is the most prevalent skin cancer amongst black and Indian/Asian populations [28, 29].

The ratio of BCC's to SCC's in our cohort was 2.03. Recent studies point to an increasing SCC incidence relative to BCC, moving the historical 4 : 1 ratio to 2.5 : 1 or even closer. Studies attributes this to a relative SCC increase in the elderly population caused by chronic exposure to UVR [30]. In our study a larger black population may be attributing to this phenomenon.

MM accounted for 6% of all skin cancers diagnosed. This is slightly higher than previous reports from the US and Australia that indicate MM to constitute approximately 4% and 2% of all skin cancers respectively [31, 32]. Previous studies in the Western Cape of South Africa reported an increased incidence of MM in Cape Town specifically when compared to other parts of South Africa [33]. The number of MM cases in our study may even be underrepresented since a large percentage of MM are diagnosed in the private sector and possibly at primary care clinics and secondary hospitals without being referred to a tertiary hospital for biopsy. Tod et al. found that 75% of all MM cases in South Africa are diagnosed in the private sector [12]. There was only one case of MM diagnosed in a patient from the black population. This lesion was located on the foot, which is in keeping with the fact that AM is the most common subtype in the black population [34]. A recent study done in the Western Cape Province reported that up to 22% of all MM diagnosed were AM and accounted for 80% of all MM diagnosed in black patients [15].

KS accounted for 7% of all skin cancers diagnosed and was the most frequent skin cancer in the black population. This finding supports data from the Northern Cape Province of South Africa that showed KS to account for 6.5% of all skin cancers and occurring more commonly in the black population [26]. When compared to data from sub-Saharan African, Nthumba et al. reported KS to represent 25% of skin cancers diagnosed in Kenya in 2008 [24]. Although HIV status for subjects was not reported in the current study HIV infection remains the biggest risk factor for developing KS in South Africa [18].

It is estimated that 18.9% of the South African population between the ages of 15–49 are HIV positive [16, 17]. The higher rate of infection in this age group is reflected by the mean age of presentation of KS in our study being 41.3 (Table 5). A study investigating the burden of cancers associated with HIV in the South African public health sector between 2004 and 2014 also demonstrated that cancer proportions were highest between the ages of 25 and 49 with the greater proportion of cancers observed in the black population. The Odds Ratio for PLHIV to develop KS in SA was reported as 134 [18]. PLHIV are also at higher risk for developing NMSC with a 5-fold increased risk of SCC [18, 19]. This may also explain the lower BCC to SCC ratio as mentioned.

KA was considered a separate entity from SCC in the study cohort and comprised of 4% of skin cancers. The true nature and its relationship to SCC continues to be controversial as some authors consider KA as a precursor of SCC, a well-differentiated SCC or an abortive malignancy with invasive potential [35]. The true incidence of KA is probably underestimated because of misdiagnosis as a SCC, underreporting or spontaneous regression [36]. The SCC/KA ratio in our cohort was 5.9. When compared to international published literature this ratio varies in studies between 2.5 : 1 and 139 : 1, most likely due to the variation in approach by pathologists [36].

The proportion of clinically suspected skin cancers that were histologically confirmed was 68%. This number compares favourably to the Skin Cancer Audit & Research Database (SCARD) of Australia which showed the percentage of new lesions tested which were malignant, during the same time period, to be 61%. SCARD is a surgical log designed for doctors treating skin malignancies in Australia with 122 562 lesions biopsied during the same time period as the current study (September 2015–August 2016) [37].

A recent publication that reported on skin biopsies and skin cancer treatment procedures in the US, showed that in 2015 only about 50% of biopsies resulted in a skin cancer diagnosis. The authors concluded that the threshold for biopsy may be decreasing, with more biopsies yielding negative results being performed [38].

The NNT for all skin cancers in our study cohort was 1.5 and is comparable to a study evaluating the US dermatologists at discriminating skin cancers that showed an NNT for all skin cancers of 2.22 [39].

The NNT for melanoma was 4, referring to the number of pigmented lesions needed to be biopsied to diagnose one melanoma. If this is compared to SCARD data from the same time period, lesions tested to find one melanoma was 5.3 [37]. Internationally published literature reported NNT numbers for melanoma to vary from 4 to 40, with lower NNT's generally being documented by dermatologists [40, 41]. Two similar studies in the United Kingdom (UK) analysing clinical diagnosis of melanoma by dermatologists reported NNT's between 2.74 and 6.3 [42, 43]. The NNT in our study though was calculated by including all pigmented lesion that were biopsied to rule out melanoma and not just melanocytic nevi, as was the case in the UK studies.

At the time of our study, dermoscopy was not routinely utilized in the diagnosis of skin cancer at the TAH dermatology OPD. Studies have shown that dermoscopy improves the diagnostic accuracy of both MM and NMSC leading to a decrease in NNT [44]. One can expect that the NNT for skin cancers has improved at the TAH dermatology OPD since, as dermoscopy is now used routinely on every skin cancer patient, although this remains to be tested.

Punch biopsy was the mostly frequently utilized modality for skin cancer biopsies and accounted for 50% of all biopsies performed. This contrasts with data from Australia that showed shave biopsies to make up 44.1% of biopsies compared to 23.4% punch biopsies during the same time frame [37]. Farberg et al. who investigated the practice patterns of the US dermatologists for biopsy of MM also reported shave biopsy (35%) to be the most commonly used method [45]. Shave biopsy offers the advantage of shorter procedure time, decreased cost, and minimal bleeding [46]. In the past shave biopsy of cutaneous lesions has been discouraged out of fear for compromising accurate diagnosis and microstaging of melanoma. Recent studies though have shown that when done correctly by a trained professional, shave biopsies are reliable and accurate in the majority of cases [47]. Training practices at the TAH may need to be reviewed to encourage shave biopsies for diagnosis of skin cancer.

## 6. Study Limitations

This was a retrospective study that relied on the completeness of pathology reports. Limiting factors were details such as duration, location, and ethnicity often being omitted. Despite providing imputated ethnicity according to surname, there is no record of Fitzpatrick skin type, which presents difficulties in deducing prevalence of certain malignancies in different skin types. It should also be kept in mind that data collection was limited to one tertiary hospital and that the study took place on a Western Cape population. The distribution of skin types in the Western Cape is very different from the rest of South Africa and there is no previous data from the Western Cape from which comparisons could be drawn. In the light of the high HIV prevalence in South Africa, the fact that HIV status of patients was not recorded leads to study limitation.

## 7. Conclusion

The study highlights the burden that skin cancer places on the health care system and provides valuable scientific data on the accuracy of skin cancer diagnosis, distribution, and patient demographics in the Western Cape Province of South Africa. The study calls for standardised reporting methods to increase the accuracy of databases and to promote and assist further research. It emphasises the need to further describe risk factors for skin cancer, to promote prevention and to improve diagnostic and management strategies.

## Figures and Tables

**Table 1:** Diagnosis of all biopsies.

Histological diagnoses (*N* = 696) *n* (%)	BCC	252 (36.21)
	SCC	87 (12.5)
	SCCI	37 (5.32)
	KS	31 (4.45)
	Melanoma	28 (4.02)
	Keratoacanthoma	21 (3.02)
	Other malignant	4 (0.57)
	Solar keratosis	6 (0.86)
	Melanocytic naevi	21 (3.02)
	Seborrhoeic keratosis	29 (4.17)
	Dermatofibroma	5 (0.72)
	Other benign	129 (18.53)
	Insufficient biopsy/no histological dx	20 (2.87)

**Table 2:** Demographics of patients with biopsy confirmed skin cancer.

Age (mean, 95% CI)		65,68 (63, 91-67, 46)
Sex (*N* = 390) *n* (%)	Male	142 (53.38%)
	Female	124 (46.62%)
Race (*N* = 390) *n* (%)	Unknown	7 (2.63)
	Mixed ancestry	34 (12.78)
	White	201 (75.56)
	Black	23 (8.65)
	Asian	1 (0.38)
	Indian	0

**Table 3:** Location of all biopsies performed.

Location of lesion (*N* = 652) *N* (%)	Face	316 (48.47)
	Leg	93 (14.26)
	Arm	53 (8.13)
	Back	53 (8.13)
	Chest	30 (4.60)
	Hand	28 (4.29)
	Neck	26 (4.00)
	Shoulders	21 (3.22)
	Foot	12 (1.84)
	Abdomen	10 (1.53)
	Other	8 (1.23)
	Buttocks	2 (0.31)

**Table 4:** Skin cancer per ethnic group.

Type of lesion	White (*n*, %)	Mixed ancestry (*n*, %)	Black (*n*, %)	Asian (*n*, %)	Unknown (*n*, %)	Total
BCC	225 (89.29)	20 (7.94)	4 (1.59)	1 (0.40)	2 (0.79)	252
SCC	74 (85.06)	6 (6.90)	6 (6.90)	0	1 (1.15)	87
SCCI	31 (83.78)	5 (13.51)	1 (2.70)	0	0	37
KS	3 (9.68)	3 (9.68)	21 (67.74)	0	4 (12.90)	31
MM	24 (85.71)	3 (10.71)	1 (3.57)	0	0	28
KA	18 (85.71)	2 (9.52)	0	0	1 (4.76)	21
Malignant other	2 (50)	2 (50)	0	0	0	4
Total	377	41	33	1	8	460

**Table 5:** Age and gender distribution for all skin cancers.

Skin cancer	*n* (%)	Age (mean, 95% CI)	Male (*n*, %)	Female (*n*, %)
BCC	252 (54.8)	68.53 (67.06–69.99)	154 (61.11)	98 (38.89)
SCC	87 (18.9)	66.9 (63.76–70.04)	54 (62.07)	33 (37.93)
SCCI	37 (8)	66.9 (63.63–70.34)	21 (56.76)	16 (43.24)
KS	31 (6.7)	41.29 (36.55–46.02)	15 (48.39)	16 (51.61)
MM	28 (6.1)	68.13 (64.41–71.85)	13 (46.43)	15 (53.57)
KA	21 (4.6)	66.46 (61.36–71.57)	11 (52.38)	10 (47.62)
Malignant other	4 (0.9)	72.32 (61.57–83.06)	2 (50)	2 (50)

## Data Availability

The data used to support the findings of this study are available from the corresponding author upon request.
